# Do integrative approaches to health contribute to self-reliance in primary healthcare? reflections from a community case study in Kerala, India

**DOI:** 10.1080/21642850.2022.2146585

**Published:** 2022-11-18

**Authors:** BS Shivanand, M Tushara, PM Unnikrishnan, Jayanna Krishnamurthy

**Affiliations:** a The University of Transdisciplinary Health Sciences and Technology (TDU), Bengaluru; b Institute of Public Health (IPH), Bengaluru; c M S Ramaiah University of Applied Sciences, Bengaluru

**Keywords:** Integrative healthcare approaches, primary healthcare, self-reliance, health system, health policy, social determinants of health

## Abstract

A pluralistic Health system provides options for people to choose appropriate healthcare approach. However, the ability to make informed decision is infleuenced by many factors. An informed decision is one of the attributes of self-reliance. In this study, through the interactions with smallholder farmers, we tried to travel through the realm of communities’ integrative practices and perceptions with a specific focus on traditional medicine concerning animal and human health. We aimed to understand what influences healthcare choices, mainly traditional medicine among people and how it contributes to self-reliance in primary healthcare. We conducted this case study in Aluva taluk of Ernakulam district in Kerala, India. Study participants were selected using the purposive sampling method and the data collected through 22 in-depth interviews and participant observation.

Integrative healthcare practice is fragmented due to variations in the evidence perception by the people. Personal experiences, social and cultural factors, and health literacy influence health decisions in practicing integrative healthcare. Therefore, while investigating a concept like self-reliance, there is a need for analytical methods to embrace experiential, textual, inherited, and incorporated forms of learning. This further helps researchers and policymakers to recommend context-specific and sustainable solutions to create self-reliant communities in primary healthcare.

## 1. Introduction

There exist practices of different healthcare approaches including traditional medicine (TM) in Kerala (Sujatha, 2007). TM is based on worldviews that consider physical, mental, social, spiritual, cultural, and ecological dimensions of health (Porter et al., 2021). The striking feature of traditional medicine is that there are different tiers of the prototype layer of medical knowledge classified as: experiential, textual, inherited, and incorporated (Oyebode et al., 2016). TM in India includes codified and non-codified systems (Oyebode et al., 2016). The codified system has acquired legitimacy and formal recognition of the practice (AYUSH). Non-codified or folk medicine is an intergenerational knowledge existing in all communities in India. This includes bone setters, birth attendants, local healers, and the use of home remedies by mothers or elders in the family for addressing common illnesses contributing to inherited and incorporated knowledge of TM (Adhikari & Paul, 2018, jan 1; Reviving local health knowledge for self-reliance in primary healthcare, 2020). Here, the iterative process of practice itself is a great source of knowledge that contributes to the use of TM (Porter et al., 2021).

Practicing TM as the first line of treatment or a combination with modern medicine is deeply ingrained in the communities of countries like India, especially in the State of Kerala (Payyappallimana, 2010), demonstrating the already existing pluralistic healthcare practices in the region. The practice of TM sometimes is because of push factors (inaccessibility, non-availability, and unaffordability of conventional health services) of the health system. In such a scenario, a healthcare choice becomes a conditioned approach for individuals rather than an approach that is chosen by them. TM is also practiced because of its close association with culture, values, beliefs, traditions, and customs. These factors strongly influence more than the medicinal value alone (Payyappallimana, 2010). Thus, the existing pluralism that provides autonomy in choosing healthcare approaches is the positive side of the people-centric approach, but there is also a danger with such choices if there is a lack of information on what to choose, how to choose, and why to choose a healthcare approach (Porter et al., 2021). Therefore, the question of informed decision is complex to address since it involves various factors (Lewis & Pignone, 2009).

Self-treatment practices consisting of TM that is adopted in families and communities sometimes lead to detrimental effects on health or delay in seeking institutional healthcare (Basappa, 2021). However, one can't undermine the experiential knowledge embedded in the community that is preventing and curing minor health conditions. ‘*Yashmin deshe to yo jaatah, Tasya tajjaushadham hitam’ (Sushruta Samhita),* which means for a person born in a biogeographical region, local herbs of that location are beneficial (Gangadharan et al., 2009). The spirit of this three millennia old proverbial statement of an Ayurveda treatise translates as a lived experience of health and nutrition of several communities in India even today, as evident in our fieldwork. The idea of nature as a remedy is embedded in people's minds within their promotive, preventive, and curative approaches to healthcare (Jayanna, 2021; Victorson et al., 2020). Most farmers in our study narrated an interwoven relationship between the local herbs, environment, animals, food, and lifestyle to prevent and treat minor health conditions and achieve control over their health and wellbeing, declaring themselves as self-reliant in primary healthcare.

The attributes of self-reliance are informed decisions, use of local resources, self-worth, and discipline (Lowe, 2003). Self-reliance is the ability of a person to manage their health conditions using locally available resources, and it is an essential strategy in the context of primary healthcare (Fonchingong & Fonjong, 2002; Weltgesundheitsorganisation, 2008). The informed decision being a necessary element of self-reliance, it plays an important role in healthcare choices. The ability to make informed decisions varies and is influenced by various factors (Lewis & Pignone, 2009). For instance, availability and accessibility of health services, perceived effectiveness, doctor-patient relationship, and social and cultural factors (Khalikova, 2020). Understanding factors that influence informed decisions may help design interventions that enable the self-reliance of people and communities.

In this study, through the interactions with smallholder farmers, we traveled through the embedded realm of farmers’ integrative healthcare approaches (those approaches that take account of body, mind, spirits, and all aspects of lifestyle) (Hu et al., 2015) with a specific focus on TM practices concerning animal and human health. With its emphasis on localization, indigenization, culture, religious practices, and context-specific solutions, TM potentially contributes to self-reliance in primary healthcare (Bodeker & Kronenberg, 2002; Savatagi et al., 2022). We aimed to understand what influences people to adopt a healthcare approach, especially TM and how they navigate their healthcare choices, and how they perceive themselves as self-reliant in primary healthcare using TM. We believe that understanding people's adaptive and pluralistic lifestyles could enhance our logical and practical approach to developing strategies that enable people to make informed decisions. This further helps to apply the concept of ‘self-reliance’ in primary healthcare using TM.

## 2. Methodology

### Study site

We conducted this case study in Aluva taluk of Ernakulam district in Kerala from November 2021 to March 2022. As per the census of 2011, the total population of Aluva is 22,428 (11,031 males and 11,397 females). The majority of the population (41.83%) self-identify as Hindus, followed by Christians (38.86%) and Muslims (18.93%). The average sex ratio of Aluva taluka is 1033 females per 1000 males. Malayalam is the native language of Aluva. Agriculture is the main occupation of the people residing in Aluva. Aluva taluka has semi-urban and rural contexts.

### Study design and selection of study participants

It is a community case study conducted using a qualitative approach. The University of Transdisciplinary Health Sciences and Technology (TDU), Bengaluru, a private university in Karnataka state in India (The University of Trans-disciplinary Health Sciences and Technology [Internet], 2022), implemented a study in Aluva. Mastitis among cattle is highly prevalent in Kerala, and also this is an economic and social concern for the farmers. The cost incurred for treating this disease using modern medicine is enormous and seems to burden the farmers. Therefore, TDU used TM using a combination of Aloe vera, calcium hydroxide, and turmeric to treat this disease, and the results were encouraging. TM use also showed many advantages in reducing the economic burden and antibiotic residue in milk and meat products. This intervention was considered an integrative approach to health since the interventions benefitted human health as well. Hence, we purposefully selected Aluva to explore if the use of traditional medicine enables the self-reliance of individuals and communities in primary healthcare. With the pre-existing list of participants provided by TDU, we traced our study participants in Aluva.

A case was defined as a community where TM interventions are practiced for treating minor health conditions in humans and animals. Individuals within the community were cases (study participants). Study participants were selected using the purposive sampling method since that provides an opportunity to select information-rich cases to investigate the phenomena of interest.

### Sampling and data collection

The sample size was determined based on the data saturation principle. Through the purposive sampling technique, we collected data from 22 cases. After 18 cases, data became repetitive, and additional three interviews were conducted to re-confirm the saturation. Hence, the total sample size became 22 cases. Two data collectors, an interviewer, and a note-taker collected the data. Both data collectors were familiar with the native Malayalam language and were not part of the community. Each interview lasted for half an hour to one hour. The observation technique was used as a supplementary to interview during the data collection process. All interviews were recorded in a recorder, and field observations were noted down in a paper. The data collectors expanded all the field notes on the day of data collection itself to use them in the analysis.

### Data collection tool

The questionnaires were developed and pilot tested prior to actual data collection. The training was provided to data collectors to understand the questionnaires and clarify their doubts before the data collection process. Questionnaires were also refined when required, mainly by adding more probes or in case the question deviated from the objective. The data captured was especially about people’s perception of health and illness, different healthcare approaches adopted by study participants and reasons to do so, and reasons for using traditional medicine.

### Data analysis

All the interviews were translated into English before exporting to software for analysis. The data sets were cross-checked for their validity. We used the QDA miner lite 2.01 version to analyze the qualitative data (QDA Miner Lite 1.^2^ Download (Free) – QDALite.exe [Internet], 2022). The thematic analysis approach was used. The themes and codes were discussed periodically with other authors and refined as and when required.

### Ethical consideration

We obtained ethical review clearance from the ethics committee for human research Trans-disciplinary Health Sciences and Technology (TDU), Bengaluru (study protocol number: TDU/IEC/11/2020/PR38). The review committee consisted of two committees scientific review committee and the ethics review committee, the former validated the scientific merit of the study, and the latter reviewed the ethical aspects of the study. The protocol was systematically reviewed and approved by both committees.

We followed the necessary ethical guidelines during the study. We provided sufficient information about the study to the participants before obtaining written consent for their participation. Potential risks and benefits were explained to the participants. We sought permission from study participants to take pictures, document their knowledge of traditional medicine, and publish the study's findings. The collected data was coded, and confidentiality was maintained throughout the study. A voluntary approach was encouraged for their participation in the study.

## 3. Results

### Characteristics of study participants

The age range of study participants was 20–80 years. The majority of the participants were Hindus 9 (∼ 40%) and Christians 9 (∼ 40%), and others belonged to Islam. The educational level ranged from 6th std to post-graduation. Of the 22 participants, 11 (50%) were male, and 11 (50%) were female. Farming was the main occupation for both male and female participants, i.e. of the total 22 participants, 21 (99%) engaged in farming, and only 1 (1%) were engaged in teaching. For details of each participant, please refer to Table 1.

**Table 1:** . Characteristics of study participants.

Sl. No	Place	Age (yrs.)	Sex	Education	Religion	Occupation
1	Choornikarna	48	F	Nursing	Christian	Teaching
2	Kudugallor	19	F	SSLC	Muslim	Farming
3	Udaymperoor	44	F	Post-graduation	Hindu	Farming
4	Angamaly	52	M	SSLC	Christian	Farming
5	Thuravoor	58	M	9th std	Hindu	Farming
6	Angamaly	47	M	Degree	Hindu	Farming
7	Aluva	31	M	SSLC	Hindu	Farming
8	Aluva	53	M	5th	Muslim	Farming
9	Choornikarna	36	M	8th	Hindu	Farming
10	Champanad	51	F	SSLC	Muslim	Farming
11	Aluva	56	M	3rd	Hindu	Farming
12	Aluva	61	M	5th std	Hindu	Farming
13	Aluva	66	F	6th std	Christian	Farming
14	Manikyamangalam	53	F	PUC	Christian	Farming
15	Manikyamangalam	62	F	3rd std	Christian	Farming
16	Manikyamangalam	68	M	PUC	Christian	Farming
17	Manikyamangalam	55	F	10th std	Hindu	Farming
18	Perumbavoor	55	F	SSLC	Muslim	Farming
19	Arakkapaddy	65	M	SSLC	Hindu	Farming
20	Manikyanmangalam	80	M	6th std	Christian	Farming
21	Mattoor	61	F	10th std	Christian	Farming
22	Aluva	61	F	6th std	Christian	Farming

### Integrative approaches toward health and wellbeing

Participants defined health as a condition free from diseases. They opined that, be it animals or humans, diseases occur due to changes in food habits, exposure to chemicals in daily life, and maintaining unhygienic living conditions. Fever, Theileria, Foot and Mouth Disease (FMD), Mastitis, Indigestion, Diarrhea, Skin infections, and Repeat bleeding were a few conditions that their cattle suffer from. Among these conditions, Mastitis was mentioned by the majority of participants. The disease Mastitis affected farmers since it led to less milk yield, high cost of treatment, and sometimes death of cattle leading to an increased burden on farmers’ health and livelihood.

As narrated by farmers, the approach to treating these conditions starts with using medicinal plants or sometimes allowing them to live in a natural environment. The decision to manage these conditions was based on their cattle's bodily reactions and the experience of living with the cattle. Some also argued that lifestyle concerns make people and animals prone to diseases. Throughout the fieldwork, farmers continue to stress that their experience in relation to geographical region, climate, and natural habitat is the major source of knowledge to manage their health and the health of domesticated cattle.

‘*A 47-year-old male participant from Angamaly village said*, “*Our cows have a lot of immunity power because of the exposure to mud and dirt. I don’t tie these cows until 6. Mostly, I bring in only those cows that have milk to tie them. We try our best to establish their relationship with nature organically. Because of all this, they don’t have big diseases.*’

The presence of internal and external drivers of change within the community to sustain animal health by using natural resources is apparent here. Thus, communities have believed and experienced a lifestyle that they think is sustainable, and they negotiate this relationship every day, bridging the gap between generations through such practices.

**Figure 1.**
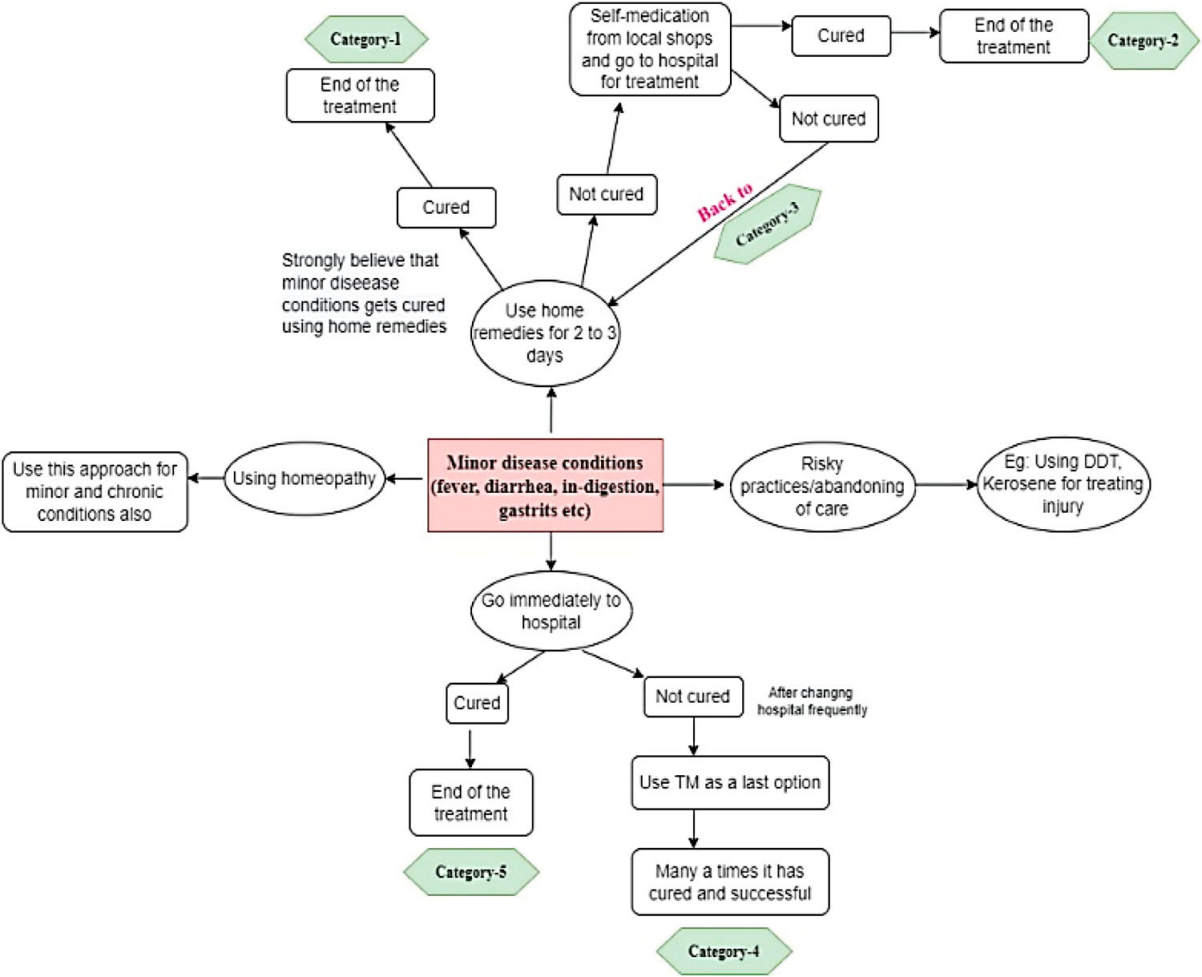
Healthcare approaches adopted by study participants.

The below diagram (Figure 1) depicts healthcare approaches used by the participants in treating minor diseases. The participants were highly dependent on three medical practices: Allopathy, Ayurveda, and Homeopathy. It was also understood that self-medication and home remedies often overlapped with the ayurvedic treatment modality. The ability to choose different treatment modalities was quite evident among the study participants, either through a trial-and-error approach or through informed decisions. As the following goes:

‘*For Mastitis, injection is not necessary, I have felt that aloe vera gave good results. Like all the rest, we haven't tried these Ayurveda medicines; rather, we rely on English medicine. We started understanding Ayurveda remedies slowly for other disease conditions. We give betel leaves and pepper for indigestion, which is quite effective*.’ *51-year-old participant, Champanad village, Aluva*.

### Healthcare approaches and their implications

Our field data indicated important perceived pros and cons of all healthcare approaches adopted by the study participants. Even though Allopathy as a medical approach was widely practiced, most participants found the influence of antibiotics harmful to the overall well-being of the cattle. Around 12 (∼ 45%) of the study participants believed that modern medicines ensured a faster cure but sustained further damage to milk production and the cattle's health status. The availability of doctors and medical practitioners in the wee hours was raised as a major issue that bothered the farmers. Only 4 (∼18%) trusted homeopathic treatment for specific diseases like Foot and Mouth Disease (FMD). All the farmers widely respected and appreciated traditional home remedies due to the ease of access, cost-effectiveness, and no side effects. However, most of them found the application of traditional medicines to be time-consuming and comparatively slower in procuring results. Within each of these approaches, different factors influenced pluralistic healthcare engagements. For instance, a statement by a participant who brought up the issues of institutional-based healthcare and stressed the need for developing skills by ourselves in handling minor issues goes as below:

‘*There are injections to dry out the wound, but not many doctors know it. If we keep calling doctors for minute issues, this deal cannot move further, and we should know certain basics. What I mean to say is, the last day a doctor came here, he gave glucose drip and gave an injection for fever, and he charged 1200 rupee as fees. Similarly, for FMD, I did not call a doctor this time; we treated on our own using the medicinal plants available in our field. (53-year-old male participant, Aluva town).*’

This statement shows that farmers’ experiences have come in handy through peer interactions and intergenerational information for their self-care approach. This knowledge also stems from local community health workers, ethnoveterinary practitioners, and also from veterinarians who have been serving the community. We can notice how they have used both allopathy and home remedies to cure the wound and discomfort of diseases like fever and FMD. For treating common ailments, they resort to remedies from their garden like *Pacha manjal (*Curcuma longa)*, mala inchi (*Zingiber wightianum***)****, vettila (Piper beetle), chengalamperanda (Cissus quadrangularis), kurumulaku (Piper nigrum), etc.,* It was quite evident in this study that they resorted to immediate treatment via the ecosystem and readily available plant products. In the light of yielding positive results, farmers reinstate those traditional medicines that enhance sustainability, prevention, and a cost-efficiency mode of treatment.

## 4. Discussion

### A struggle to find the meaning of informed decision

Attributes of self-reliance were embedded within the integrative healthcare approaches of study participants. The integrative approaches that are taken by the participants highlight the existence of a medically pluralistic system. However, these approaches were most often through a ‘trial and error’ technique. When choosing between evidence-based and practice-based medicine, respondents tend to choose efficiency over authenticity since they are more inclined towards results than science. From our study, we interpret that there was an internal struggle among study participants between understanding their bodies and the lifestyle they were living (Gabre et al., 2019) to preserve their health. This internal struggle was due to the mismatch between the values they believed in and the practice they were doing. Their trial-and-error approach seems out of tension to address a particular health problem. Within this struggle, they were also able to bring a new pathway to managing disease conditions. This observation was similar to a review by Lakshmi et al. (2015) that described how disagreements within a family and community exist that trigger the friction of the decision process in seeking healthcare due to the accessibility of pluralistic healthcare approaches (Lakshmi et al., 2015). Therefore, the act of creating meaning to resources and decisions came from the struggle participants underwent by negotiating with their beliefs, values, cultural relevance, and their health literacy (Martínez et al., 2021).

Historical developments in medicine show that medicine was borne out of human necessity, but in the current situation, we have multiple options of healthcare approaches (Park, 2013; Payyappallimana, 2010; Rudra et al., 2017). Thus, the health system has moved from a mere being disease or target-specific approach to a comprehensive approach to health and wellbeing by providing multiple healthcare options. Therefore, now it is not about necessity; rather, it is about the right choice through the crucial informed decision. However, the decision ability remains complex and ambiguous (Sundararajan et al., 2020). To take advantage of the strengths of different healthcare approaches, there is a need to increase the health literacy of the people to make them understand the importance of integrative healthcare approaches, and that could enhance their ability to make informed choices.

### What does self-reliance mean for the study participants?

The meaning of self-reliance given by participants exists in the negotiation process than in a straightforward response to a health problem. This negotiation originated out of their experiential knowledge that allowed participants to understand their bodies in relation to their surrounding environment. Thus, the ability of self-awareness of the body and its reactions to a disease condition is a great source for making choices related to healthcare (Bullington, 2006). If self-awareness emerged from aligning the belief system and the practices of study participants, it contributed significantly to an informed decision, thereby enabling their self-reliance. Thus, the efficacy and legitimacy of a healthcare approach are evaluated by people depending upon how a modality of treatment is able to consider their personal views while addressing a health problem. This finding aligns with the study conducted by Khalikova (2019) that mentions personal and family experiences as a great source for determining the efficacy and legitimacy of a healthcare approach (Khalikova, 2020). Thus, we interpret that self-reliance is a social and complex construct that involves many factors at different levels in its manifestation. This observation is similar to a review by Savatagi et al. in 2022 (Savatagi et al., 2022).

### Need for emphasizing self-reliance as a strategy in the context of primary healthcare

Apart from existing confusion in the choice of healthcare, most of the time, farmers found it difficult to get a qualified medical professional/veterinarian in case of emergency (Muraleedharan & Chandak, 2022). That is also one of the prime reasons to resort to TM. In addition, increased antibiotic use, increased health expenditure, and repeated infections pushed farmers to rely on their resources as a primary response to their health problems (Rudra et al., 2017). The existing understanding of farmers about their place, context, and the local ecosystem was a pull factor that enabled them to use their natural resources.

With the renewed interest in primary healthcare among policymakers, there is a need to bring innovative and integrative strategies that are people-centric, context-specific, and can be applied at the grassroots level to achieve universal health coverage (World Health Organization, 2007). While we try addressing push factors, we also need to strengthen pull factors to apply and practice self-reliance logically. Although integrative healthcare approaches provide more autonomy in choosing treatment modalities; however, this choice should be made strategically rather than arbitrarily. The increased voice towards a people-centered health system calls for those strategies where people’s abilities are built and given more autonomy to choose healthcare approaches based on their social, cultural, ecosystem, and other contextual factors as they influence their healthcare decisions (Sheikh et al., 2014).

### Strengths and limitations

Our study discussed the importance and complexity of informed decisions while adopting integrative healthcare approaches and in the manifestation of self-reliance in primary healthcare. We have taken people's perspectives in understanding reasons to utilize integrative healthcare approaches, including TM. However, perspectives from healthcare practitioners/providers (veterinarians, medical officers, community health workers) could have brought perspectives of healthcare providers, which we acknowledge as a limitation of our study.

## Conclusion

The practice of pluralistic and integrative approaches is fragmented due to variations in the evidence perception by the people. An amalgamation of personal experience, social and cultural factors and health literacy sourced from community health workers, family members, healers, and other health service providers help people to make informed decisions. To address the existing trial and error approach of a healthcare choice, there is a need to build the competency of both healthcare providers and consumers. This, in turn, helps families and communities to use integrative healthcare approaches through informed decisions. While investigating a concept like self-reliance, the analytical methods must focus on local worldviews and lived experience and their inner meaning. This approach should also embrace experiential, textual, inherited, and incorporated forms of learning and thereby help researchers to unpack the complexity of the decision process in using integrative healthcare approaches. This further helps policymakers to recommend solutions that are context-specific and sustainable to create self-reliant communities in primary healthcare. In addition, emphasizing localization, preserving natural resources, and protecting the interrelation between human, animal, and local ecosystems continued to remain as core elements of self-reliance while achieving health and wellbeing.

## Authors’ contributions

All authors contributed significantly to the development of the manuscript. SBS (first author) and TM (second author) were involved in data collection, transcription, and analysis. TM supported SBS right from the drafting till the completion of the article. UPM (third author) and JK (fourth author) critically reviewed the article in every step and guided throughout the process.

## Open Scholarship

This article has earned the Center for Open Science badges for Open Data and Open Materials through Open Practices Disclosure. The data and materials are openly accessible at https://doi.pangaea.de/10.1594/PANGAEA.915881.
